# Supplementary material for: Reconciling Mining with the Conservation of Cave Biodiversity: A Quantitative Baseline to Help Establish Conservation Priorities
Source: PLoS One. 2016 Dec 20;11(12):e0168348. doi: 10.1371/journal.pone.0168348 (PMC5173368; doi:10.1371/journal.pone.0168348)
Supplement: S1 Dataset — (ZIP) [file pone.0168348.s002.zip › Taxa/Serra Norte/SN_2013/N5S/Lista N5S-71.pdf]

CAVIDADE N5S-0071

Classe	Ordem	Fam/Outros	Gên/Outros	Espécie	S-U	U-S	Zon.
Annelida	Oligochaeta			jovem		1	E
Arachnida	Acari	Anystidae	*Erythracarus*	sp.1	X		E
Arachnida	Amblypygi	Phrynidae	*Heterophrynus*	*longicornis*	1	1	E
Arachnida	Araneae	Mysmenidae	*Microdipoena*	sp.1	X	X	E
Arachnida	Araneae	Pholcidae	*Mesabolivar*	aff. *cambridgei*		X	E
Arachnida	Araneae	Pholcidae	*Mesabolivar*	sp.1	X	X	E
Arachnida	Araneae	Salticidae		jovem		X	E
Arachnida	Araneae	Theridiidae	*Dipoena*	sp.2		X	E
Arachnida	Araneae	Theridiidae	*Echinotheridion*	sp.1	X		E
Arachnida	Araneae	Theridiidae	*Theridion*	sp.2		X	E
Arachnida	Araneae	Theridiosomatidae	*Plato*	sp.1		X	E
Arachnida	Araneae	Theridiosomatidae	*Plato*	sp.1		X	E
Arachnida	Pseudoscorpiones	Chthoniidae	*Pseudochthonius*	sp.1	X		E
Insecta	Blattodea			jovem	1		E
Insecta	Coleoptera	Carabidae		sp.3	X		E
Insecta	Coleoptera	Dytiscidae		sp.2		X	E
Insecta	Coleoptera	Staphylinidae	Pselaphinae	sp.9		X	E
Insecta	Diptera	Cecidomyiidae	Cecidomyiinae	sp.		X	E
Insecta	Diptera	Culicidae	*Culex*	sp.		X	E
Insecta	Diptera	Psychodidae	Phlebotominae	sp.		X	E
Insecta	Diptera	Simuliidae		sp.		X	E
Insecta	Diptera	Tipulidae	Tipulinae	sp.	X	X	E
Insecta	Heteroptera	Reduviidae	Reduviinae	sp.	X	1	E
Insecta	Heteroptera	Veliidae		sp.		X	E
Insecta	Hymenoptera	Formicidae	*Dolichoderus*	aff. *bispinosus*		X	E
Insecta	Hymenoptera	Formicidae	*Paratrechina*	sp.2		X	E
Insecta	Isoptera	Termitidae	*Nasutitermes*	sp.		X	E
Insecta	Lepidoptera	Galacticidae		sp.		X	E
Insecta	Lepidoptera	Noctuoidea		sp.5		2	E
Insecta	Orthoptera	Phalangopsidae	*Paraclodes*	sp.	1	3	E
Insecta	Psocoptera	Epipsocidae	*Epipsocus*	sp.1	X	X	E
Insecta	Thysanura	Nicoletiidae		jovem	X		E
